# Expression of codon optimized genes in microbial systems: current industrial applications and perspectives

**DOI:** 10.3389/fmicb.2014.00021

**Published:** 2014-02-04

**Authors:** Claudia Elena, Pablo Ravasi, María E. Castelli, Salvador Peirú, Hugo G. Menzella

**Affiliations:** Genetic Engineering and Fermentation Technology, Facultad de Ciencias Bioquímicas y Farmacéuticas, Universidad Nacional de Rosario-ConicetRosario, Argentina

**Keywords:** synthetic biology, gene design, codon optimization, strain engineering, microbial systems

## Abstract

The efficient production of functional proteins in heterologous hosts is one of the major bases of modern biotechnology. Unfortunately, many genes are difficult to express outside their original context. Due to their apparent “silent” nature, synonymous codon substitutions have long been thought to be trivial. In recent years, this dogma has been refuted by evidence that codon replacement can have a significant impact on gene expression levels and protein folding. In the past decade, considerable advances in the speed and cost of gene synthesis have facilitated the complete redesign of entire gene sequences, dramatically improving the likelihood of high protein expression. This technology significantly impacts the economic feasibility of microbial-based biotechnological processes by, for example, increasing the volumetric productivities of recombinant proteins or facilitating the redesign of novel biosynthetic routes for the production of metabolites. This review discusses the current applications of this technology, particularly those regarding the production of small molecules and industrially relevant recombinant enzymes. Suggestions for future research and potential uses are provided as well.

## INTRODUCTION

Microorganisms are at the core of the production of pharmaceuticals, industrial enzymes, and fine chemicals. In many cases, heterologous expression of genes is required to meet commercial-level demands of target proteins and/or metabolites. In this context, variation in codon usage is considered as one of the major factors affecting protein expression levels, since the presence of rare codons can reduce the translation rate and induce translation errors with a significant impact on the economics of recombinant microbe-based production processes (Ikemura, 1981; Gustafsson et al., 2004). The generation of massive genome sequencing data and cost-effective custom DNA synthesis are foundational technologies for synthetic biology, an emerging discipline that aims to create novel organisms containing designer genetic circuits for the production of drugs, industrial enzymes, biofuels, and chemicals (Endy, 2005; McDaniel and Weiss, 2005; Heinemann and Panke, 2006; Leonard et al., 2008). These circuits are built from standard biological parts, including vectors, promoters, ribosomal binding sites (RBSs), transcriptional terminators, and other gene expression regulatory elements. These parts were initially borrowed from nature and nowadays engineered, to adapt their performance to a particular application, or combined to create sophisticated devices (Shiue and Prather, 2012).

Over the past decade, synthetic biology has contributed to significantly reduce the cost of many products manufactured in microbial systems where only one gene needs to be over-expressed. In many cases, the production of a target protein can be boosted by several orders of magnitude by replacing a native sequence with its optimized counterpart (Gustafsson et al., 2004, 2012). This seemingly simple adjustment is of remarkable importance, since many of these products are now traded as commodities and thus there is a continuous need to reduce manufacturing costs in order to remain competitive in the global markets (Menzella, 2011). The ambitious next step of synthetic biology is to further reduce the cost and time involved in developing recombinant organisms by using pre-assembled parts that provide stable, predictable protein expression (Dellomonaco et al., 2010; Nielsen and Keasling, 2011).

So far, most of the progress made in synthetic biology was achieved in *Escherichia coli*, a preferred host for the production of recombinant proteins because it combines fast growth rate, inexpensive fermentation media and well understood genetics (Burgess-Brown et al., 2008; Welch et al., 2009; Menzella, 2011). However, efforts have been recently expanded to other hosts including *Streptomyces* species (Medema et al., 2011), *Corynebacterium glutamicum *(Becker and Wittmann, 2012), yeast (Krivoruchko et al., 2011; Siddiqui et al., 2012; Furukawa and Hohmann, 2013), and algae (Wang et al., 2012; Gimpel et al., 2013). This expanded landscape seeks to take advantage of the natural capabilities to synthesize precursors and cofactors required to produce a particular target, exploit secretion abilities, or utilize natural tolerance to over-accumulated metabolites (Zhu et al., 2012). In this review we summarize the current state of the technology for the expression of codon optimized genes in microbial systems. Examples of its application for the production of small molecules and recombinant enzymes of industrial interest are presented, and suggestions for future research and uses are provided.

### GENE DESIGN

Choosing a gene for optimal expression requires selection from a large number of sequences. For example, a protein with an average size of 30 kDa may, in theory, be encoded by 10^100^ possible DNA sequences (Welch et al., 2009). Historically, two approaches have been used for codon optimization. The first, designated “one amino acid-one codon,” uses the most abundant codon of the host to encode all occurrences of a given amino acid in the optimized sequence (Fuglsang, 2003; Gao et al., 2004; Supek and Vlahovicek, 2004; Villalobos et al., 2006; Feng et al., 2010; Marlatt et al., 2010; Wang et al., 2010). This simple strategy, the most popular in the early days of gene synthesis technology, has a major drawback: a strongly transcribed mRNA from a gene with this design will contain a high concentration of a subset of codons, resulting in an imbalance in the tRNA pool, which in turn may reduce growth due to tRNA depletion (Gong et al., 2006; Villalobos et al., 2006).

The second approach, named “codon randomization,” uses translation tables based on the frequency distribution of the codons in an entire genome or a subset of highly expressed genes. These tables attach weights to each codon, thus codons are assigned randomly with a probability given by the weights (Kodumal et al., 2004; Jayaraj et al., 2005; Menzella et al., 2005; Welch et al., 2009; Wang et al., 2010). This strategy was shown to be superior and was quickly adopted by the synthetic biology community. In addition to improving the yield of the desired product, the “codon randomization” strategy offers many further advantages. For example, flexibility in codon selection facilitates gene design by avoiding: (i) repetitive elements that may lead to gene deletions; (ii) internal RBSs, polyadenylation signals, or transcriptional terminators; (iii) secondary mRNA structures (Luisi et al., 2013); and (iv) by facilitating elimination of unwanted restriction sites to assist the assembly of larger constructs (Villalobos et al., 2006). Several large-scale systematic studies describing variations on this strategy have been conducted in recent years to provide data on the effect of sequence variables (Kudla et al., 2009; Welch et al., 2009; Allert et al., 2010).

Besides codon optimization, other parameters need to be considered to design a gene for efficient translation, including the global GC content (Gustafsson, 2009), local context of a given codon (Villalobos et al., 2006), the presence of mRNA sequence motifs (Pertzev and Nicholson, 2006), and the sequence of the region including the first 10 codons (Goodman et al., 2013). Many web-based free softwares, with features ranging from basic to advanced, were created for gene design during the last decade. Examples include: DNA Works (Hoover and Lubkowski, 2002), GeMS (Jayaraj et al., 2005); Optimizer (Puigbo et al., 2007); Synthetic Gene Designer (Wu et al., 2006); and Gene Designer (Villalobos et al., 2006). Currently, the majority of synthetic DNA suppliers (including GenScript, DNA2.0, GeneArt and Genewiz) offer sequence optimization services using proprietary algorithms at no additional cost.

### PARTS AND VECTORS

The application of synthetic DNA technology in engineered microorganisms is not restricted to redesigned genes. Classic expression vectors widely used in strain engineering derive from natural sources and were never optimized for robust production. Recently, great interest has arisen in the systematic engineering and standardization of gene expression parts such as promoters, translation initiation signals, transcriptional terminators, selectable markers, and replication origins to allow fast and predictable combination of these elements.

Some applications, such as metabolic engineering, require optimal levels of each enzyme to maximize production. This is typically achieved by modulating gene expression by, for example, varying transcription or translation levels. Synthetic biology can offer collections of promoters and RBSs capable of providing different levels of gene expression for this purpose (Boyle and Silver, 2012; Meng et al., 2013; Vogl et al., 2013). So far, most of the available promoters have been taken from the natural sequences driving the expression of highly expressed genes. Typical examples are the widely used AOX promoter from *Pichia pastoris *(Tschopp et al., 1987) for yeast and the bacteriophage *T7* promoter for *E. coli* (Studier and Moffatt, 1986), which provide high transcription levels. Nowadays, synthetic promoter libraries for tunable gene expression are available for many industrially relevant microorganisms including *E. coli* (Wu et al., 2013), *P. pastoris* (Hartner et al., 2008; Ruth et al., 2010; Vogl et al., 2013), *C. glutamicum *(Yim et al., 2013), and *Bacillus subtilis *(Hansen et al., 2009). Likewise, synthetic RBSs can be used to regulate gene expression (Basu et al., 2005; Pfleger et al., 2006). Furthermore a novel method for automatic design of artificial RBSs to control gene expression has been recently described, expanding the toolbox of artificial sequences to be used in custom genetic circuits (Salis et al., 2009).

Despite current efforts, accurate predictions of the response of any given promoter or RBS have often remained elusive. It is possible that unknown interactions among isolated components may significantly affect the optimal level of gene expression needed to achieve a particular flux through a biosynthetic pathway (Keasling, 2012). In a recent work, Kosuri et al. (2013) provided an alternative strategy to screen the behavior of gene expression regulatory elements. They synthesized 12,563 combinations of common promoters and RBSs and simultaneously measured DNA, RNA, and protein levels from the entire library. They found that RNA and protein expression were within twofold of expected levels 80 and 64% of the time, respectively, and that the worst 5% of constructs deviated from prediction by 13-fold on average, which could hinder large-scale genetic engineering projects. This comprehensive study provides a means to test standard part combinations to optimize production of a particular target molecule.

**FIGURE 1:**
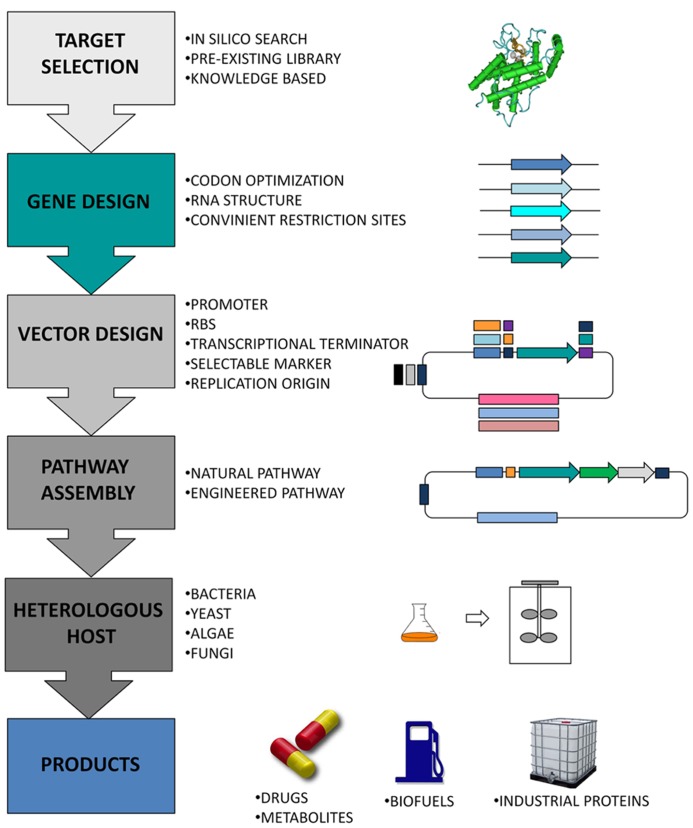
**Schematic representation of a synthetic biology network applied to the design of novel bio-based parts and devices, as well as to the engineering of existing natural biological systems for the development of target products**.

Genes are usually introduced into production microorganisms using plasmid vectors (**Figure 1**). Synthetic biology provides the means to speed up this process by using designer plasmid vectors, where all the components are synthesized with standard formats to facilitate exchange and testing of parts, as well as the assembly of multi-gene constructs (Leonard et al., 2008; Shetty et al., 2008). Several designs for the construction of synthetic plasmids and for the assembly of parts have been proposed (Menzella et al., 2005, 2007; Reisinger et al., 2006; Shetty et al., 2008; Sarrion-Perdigones et al., 2011). The most popular format among the synthetic biology community was created by Knight and co-workers (Shetty et al., 2011). They proposed the BioBrick standard, where all parts are flanked by a common set of restriction sites that allow the joining, combination, and rapid assembly of genetic parts to create functional gene expression units.

So far, most of the work to create synthetic vectors reported in the literature has been done in *E. coli*. Recently, we created a plasmid-based platform for the rapid engineering of *C. glutamicum*, a microorganism of great industrial interest. The approach uses reporter genes to examine and classify promoters and RBSs and permits the easy assembly of operons and genes clusters for co-expression of heterologous genes to facilitate metabolic engineering. Similarly, Constante and co-workers described a platform to engineer eukaryotic hosts by using the BioBrick principle. Interestingly, the system contains a variety of novel parts and implements a recombinase-mediated DNA insertion, allowing chromosomal site-directed exchange of genes in eukaryotic cell lines (Constante et al., 2011).

### PRACTICAL APPLICATIONS

The list of products obtained by the expression of codon optimized genes in microorganisms is constantly growing and includes biofuels, pharmaceuticals, novel bio-based materials and chemicals, industrial enzymes, amino acids, and other metabolites (**Table 1**).

**Table 1:** Expression of redesigned genes of industrial interest in microbial systems.

Application	Product	Host	Titer	Reference
Biofuels	Short-chain alcohols			
	Isopropanol	*E. coli*	4.9–13.6 g/L	Hanai et al. (2007)
	Butanol	*E. coli*	1.2 g/L	Inui et al. (2008)
		*E. coli*	4 g/L	Bond-Watts et al. (2011)
	Fatty acid derivatives
	C12–C18 fatty acid ethyl esters	*E. coli*	700 mg/L	Steen et al. (2010)
	Isoprenoids
	Bisabolene	*E. coli/S. cerevisae*	>900 mg/L	Peralta-Yahya et al. (2011)
Biopolymers	Polyhydroxybutyrate	*S. cerevisiae*	180 mg/L	Kocharin et al. (2013)
Chemical precursors	Methyl halides	*S. cerevisiae*	860 mg/L	Bayer et al. (2009)
Industrial enzymes	Cellulases	*S. cerevisae*	NR ^*^	Heinzelman et al. (2009)
	Phytases	*A. orizae*	NR ^*^	Lichtenberg et al. (2011)
		*P. pastoris*	12.2 g/L	Xiong et al. (2006)
	Cutinases	*P. pastoris*	NR ^*^	Liu et al. (2009)
	Lignocelluases	*P. pastoris*	1–6 g/L	Mellitzer et al. (2012)
	Lipases	*P. pastoris*	4 g/L	Chang et al. (2006)
	Prochimosin	*E. coli*	20 g/L ^**^	Menzella (2011)
	Steryl glycosidase	*E. coli*	NR ^*^	Aguirre et al. (2013)
Metabolites	L-amino acids	*C. glutamicum*	30–100 g/L	Becker and Wittmann (2012)
Therapeutics	Artemisinic acid	*S. cerevisae*	25 g/L	Paddon et al. (2013)
	Polyketide precursors	*E. coli*	Up to 90 mg/L	Menzella et al. (2007,2010)
	Multiepitope antigenic proteins	*E. coli*	NR ^*^	Talha et al. (2010), de Souza et al. (2013)

* Not Reported;

** Inclusion bodies.

Production of novel biofuels is one of the most attractive applications for synthetic biology. Fuels like ethanol, biodiesel, butanol, and terpenoid compounds are currently produced using engineered microbes (**Table 1**). In fact, the main obstacle for the production of these molecules at commercial level is the development of robust microbes and processes (Fischer et al., 2008). Synthetic biology provides tools to achieve optimal expression of pathway genes to ensure the efficient conversion of feedstock materials to target molecules, which is critical to the success of any metabolic engineering strategy. There has been considerable progress recently in the production of different biofuels, and some of the processes have reached promising yields. Hanai and co-workers combined enzymes from *Clostridium acetobutylicum* (Thl, CtfAB, and ADC), *Clostridium beijerinckii* (ADH), and *E. coli* (AtoAD) to assemble a fermentative pathway in *E. coli* that resulted in production of isopropanol at titers ranging from 4.9 to 13.6 g/L (Hanai et al., 2007). Butanol production was achieved in *E. coli* using the biosynthetic pathway from *C. acetobutylicum *and other related clostridial species, reaching titers up to 1.2 g/L (Inui et al., 2008). This was further improved to more than 4 g/L of butanol production by replacing enzymes that are naturally reversible with those that drive the reaction toward butanol formation, expressed from codon optimized genes from different bacterial species (Bond-Watts et al., 2011).

Fatty acid derivatives are other promising biofuel candidates, due to their high energy density and low water solubility. Stenn et al. engineered *E. coli* to produce C_12_–C_18_ fatty acid ethyl esters (FAEEs) directly from glucose at a titer of ~700 mg/L (Steen et al., 2010). Five engineering strategies were combined to achieve this titer, including the elimination of the β-oxidation pathway and the expression of several synthetic genes from different microorganisms. Monoterpene and sesquiterpene hydrocarbons such as limonene, pinene, and farnesene, are isoprenoid compounds with promising fuel applications that have been produced in *E. coli* and *S. cerevisiae*. Expression in *E. coli* of a codon-optimized bisabolene synthase from the fir tree *Abies grandis*, in conjunction with the introduction of an optimized heterologous mevalonate pathway, resulted in sesquiterpene bisabolene production of 900 mg/L.

A *S. cerevisiae* strain that overproduces farnesyl pyrophosphate also gave bisabolene titers higher than 900 mg/L using the same bisabolene synthase (Peralta-Yahya et al., 2011). The mevalonate pathway expression was further improved in *E. coli* by introducing codon-optimized versions of the mevalonate kinase and phosphomevalonate kinase genes after they were identified as potential pathway bottlenecks (Redding-Johanson et al., 2011).

Codon optimized genes have been extensively used to produce pharmaceuticals in microbial platforms. Polyketides are a class of natural products with a high number of well-established clinical applications. The development of a variety of methods for polyketide synthases (PKS) engineering (Menzella and Reeves, 2007; Peiru et al., 2009, 2010) led to a pioneer synthetic biology project conducted at Kosan Biosciences. The goal was to obtain polyketide precursors for the synthesis of novel drugs. First, a generic design for type I PKS genes was created to enable easy assembly and expression of chimeric enzymes (Kodumal et al., 2004; Menzella et al., 2005). The sequences of the synthetic genes were then redesigned with custom made software to optimize codon usage in order to maximize expression in *E. coli *and provide a standard set of restriction sites to allow combinatorial assembly into unnatural enzymes. Next, more than three million bases of PKS genes were tested to validate the platform. These efforts produced a variety of novel valuable compounds (Menzella et al., 2007; Menzella et al., 2010).

Another remarkable contribution of synthetic biology is the microbial production of artemisinin, a sesquiterpene endoperoxide used to treat malaria (Paddon et al., 2013). This compound is naturally produced by the plant *Artemisia annua*, but the production of plant-derived artemisinin is expensive; which limits its access to many patients. Recently, Paddon and coworkers engineered strains of *S. cerevisiae* for production of artemisinic acid, a precursor of artemisinin by fermentation. The simultaneous co-expression of synthetic genes provided an efficient biosynthetic route to artemisinic acid, with fermentation titers of 25 g/L.

Production of proteins for therapeutic use also takes advantage of the use of synthetic genes; a comprehensive review describing progress in this field has been recently published by Mitchell (2011). An elegant synthetic biology approach was used to create designer antigenic proteins for immunoassay-based diagnosis. By designing synthetic genes encoding tandem combinations of epitopes joined by flexible peptide linkers, chimeric proteins were obtained for the detection of antibodies in sera with higher sensitivity and specificity (Talha et al., 2010; de Souza et al., 2013).

The global market for industrial enzymes exceeded $4 billion in 2012 and is therefore an attractive target for cost reduction using synthetic biology tools (Zhou et al., 2004; Menzella, 2011). The use of codon optimized genes allowed notable increases in the production of many enzymes in a variety of hosts, including cellulases in *S. cerevisiae* (Heinzelman et al., 2009), phytases in *Aspergillus oryzae* (Lichtenberg et al., 2011), cutinases (Liu et al., 2009), lignocellulases (Mellitzer et al., 2012), and**lipases (Chang et al., 2006)**in *P. pastoris *and calf prochymosin in *E. coli* (Menzella, 2011). In the last example, a strain developed in our laboratory harboring a codon optimized gene produced 70% more prochymosin than that obtained with the wild type sequence, with the concomitant reduction in production costs.

In addition to the contribution to achieve more competitive production processes, synthetic genes provide an attractive alternative for the discovery of enzymes for new applications. For example, in order to search for thermostable enzymes to hydrolyze steryl glucosides (major contaminants of oil-derived biodiesel), we screened a library of archeal genes by retrieving the sequences *in silico*, synthesizing codon optimized genes for expression in *E. coli* and assessing their activity against the target. The approach was very successful and resulted in excellent candidates for industrial use (Aguirre et al., 2013). Other products of commercial interest recently obtained from strains carrying codon optimized genes include L-amino acids in *C. glutamicum* and *E. coli *(Becker and Wittmann, 2012), and polyhydroxybutyrate and methyl halides in *S. cerevisiae *(Bayer et al., 2009; Kocharin et al., 2013).

## CONCLUSION AND FUTURE PERSPECTIVES

The benefits of using codon optimized genes in industrial biotechnology have been extensively demonstrated during the past decade and this technology is being rapidly adopted by strain developers in order to remain competitive in the current market. In the examples presented here, just one or a few synthetic genes need to be introduced into a host to generate novel products or to dramatically reduce the cost of producing existing ones. The cost of synthetic genes has been constantly decreasing during the last decade; and technologies to assemble large fragments of DNA and to make multiple simultaneous changes to wild type genomes are becoming available (Montague et al., 2012). Thus, we can envision a future where custom-made microorganisms can be designed for a particular application (Gibson et al., 2010).

One of the fields where these new technologies can make a dramatic contribution is the production of commodity chemicals in microbes. Initial steps toward this ambitious goal have already been taken by industry. For example, an *E. coli* strain has been engineered to produce 1,3-propanediol, where in addition to the introduction of the pathway for the production of this target from glycerol, several changes were made in the genome to increase the final yield (Nakamura and Whited, 2003).

Although tremendous progress has been made, in order to fully harness the potential of synthetic biology we need a deeper understanding of the underlying molecular principles of living systems and further development of bioinformatic tools to assist in the modeling of synthetic genomes behavior. These advances are expected to arrive from the interactions among many scientific disciplines.

## Conflict of Interest Statement

The authors declare that the research was conducted in the absence of any commercial or financial relationships that could be construed as a potential conflict of interest. The Associate Editor declares that despite being affiliated to the same institution as the authors, the review process was handled objectively and no conflict of interest exists.
